# Step-down versus outpatient psychotherapeutic treatment for personality disorders: 6-year follow-up of the Ullevål personality project

**DOI:** 10.1186/1471-244X-14-119

**Published:** 2014-04-23

**Authors:** Bjørnar T Antonsen, Ole Klungsøyr, Anne Kamps, Benjamin Hummelen, Merete S Johansen, Geir Pedersen, Øyvind Urnes, Elfrida H Kvarstein, Sigmund Karterud, Theresa Wilberg

**Affiliations:** 1 Institute for Clinical Medicine, University of Oslo, Oslo, Norway; 2 Department of Research and Development, Clinic for Mental Health and Addiction, Oslo University Hospital, Oslo, Norway; 3 Department of Psychiatry, Lovisenberg Diaconal hospital, Oslo, Norway; 4 Department of Personality Psychiatry, Clinic for Mental Health and Addiction, Oslo University Hospital, Oslo, Norway

**Keywords:** Personality disorder, Psychotherapy, Level of care, Long-term follow-up

## Abstract

**Background:**

Although psychotherapy is considered the treatment of choice for patients with personality disorders (PDs), there is no consensus about the optimal level of care for this group of patients. This study reports the results from the 6-year follow-up of the Ullevål Personality Project (UPP), a randomized clinical trial comparing outpatient individual psychotherapy with a long-term step-down treatment program that included a short-term day hospital treatment followed by combined group and individual psychotherapy.

**Methods:**

The UPP included 113 patients with PDs. Outcome was evaluated after 8 months, 18 months, 3 years and 6 years and was based on a wide range of clinical measures, such as psychosocial functioning, interpersonal problems, symptom severity, and axis I and II diagnoses.

**Results:**

At the 6-year follow-up, there were no statistically significant differences in outcome between the treatment groups. Effect sizes ranged from medium to large for all outcome variables in both treatment arms. However, patients in the outpatient group had a marked decline in psychosocial functioning during the period between the 3- and 6-year follow-ups; while psychosocial functioning continued to improve in the step-down group during the same period. This difference between groups was statistically significant.

**Conclusions:**

The findings suggest that both hospital-based long-term step-down treatment and long-term outpatient individual psychotherapy may improve symptoms and psychosocial functioning in poorly functioning PD patients. Social and interpersonal functioning continued to improve in the step-down group during the post-treatment phase, indicating that longer-term changes were stimulated during treatment.

**Trial registration:**

NCT00378248.

## Background

Although psychotherapy is a cornerstone in the treatment of personality disorders (PDs), there is little empirical evidence regarding how different levels of psychotherapeutic care may affect treatment outcome [1-3]. Gunderson et al. [4] described “level of care” as a multi-dimensional construct that includes a variety of aspects, such as containment, intensity, the structure and duration of treatment, and cost. From an organizational perspective, the term “level of care” usually refers to whether treatment is offered as inpatient hospitalization, day hospital or outpatient treatment, regardless of the duration and intensity of therapy or whether it is conducted in an individual or group format. During the past couple of decades, several studies have demonstrated that therapies offered in outpatient, day hospital, and inpatient settings have a positive effect on patients with PDs [2,5,6]. However, few studies have directly compared different treatment settings. Recently, three Dutch multi-center studies compared treatments at different levels of care for patients with PDs. Their results suggested that more intensive treatment in day hospital or inpatient settings had beneficial outcomes compared with outpatient treatment [7-9]. However, these conclusions should be considered preliminary, as the allocation of patients was not based on randomization but on the expert opinions of clinicians.

In Europe in particular, treatment models have been developed that combine various levels of care for patients with PDs, known as step-down programs. Such programs consist of an initial intensive treatment in a day hospital or inpatient setting, followed by outpatient psychotherapy with a corresponding reduction in treatment intensity. Existing step-down programs tend to emphasize various group therapies or the combination of individual and group psychotherapy, and the treatment length is typically long-term [10,11]. Many functionally impaired patients with PDs are in need of long-term therapy and a comprehensive and integrative treatment approach [12]. Promising evidence suggests that step-down models have the potential to meet such needs for patients with severe PDs, particularly borderline PD [13-18]. However, only one step-down model has been tested in a randomized design. Fonagy and Bateman [13,14] found there were superior effects from an 18-month mentalization-based day treatment followed by outpatient group psychotherapy for another 18 months, compared with treatment as usual. Differences in outcome were still significant at 8-years follow-up [15]. So far, no randomized study has compared a step-down model with outpatient individual therapy, which appears to be the most realistic therapeutic alternative in Western societies.

Despite the assumption that patients with severe PDs might benefit from long-term treatment, there have been few randomized studies of therapies lasting more than 1 year for patients with severe PDs [14,19,20]. Also, while several studies have shown that treatment gains may be maintained for 1 or 2 years after treatment, few studies have investigated the long-term course of symptoms after treatment [15,17,21]. One might assume that, for a majority of patients, systematic psychotherapy would contribute to more adaptive personality functioning and increased resistance to future challenging life circumstances. However, naturalistic follow-along studies have revealed considerable variation over time in the clinical course of patients with PDs [22-25]. Therefore, we need more studies evaluating how treatment at various levels of care might influence the long-term course of symptoms and social adjustment.

The Ullevål Personality Project (UPP) is a randomized clinical trial (RCT) designed to investigate the effect of two treatment conditions: (1) a step-down program comprising short-term day hospital treatment followed by a combination of long-term group and individual psychotherapy for a maximum of 4 years, or (2) outpatient individual psychotherapy. The results of the 8 month, 18 month and 3 year evaluation have been reported previously [26-28]. The initial hypothesis was that the step-down treatment program would yield better clinical outcomes than outpatient individual psychotherapy. However, despite the more extensive format of the step-down treatment, results from the 18-month and 3-year follow-up studies indicated that step-down treatment was not more effective than outpatient individual psychotherapy. In fact, there was a trend towards better results with the outpatient treatment; in particular, psychosocial functioning from 18 month to 3 years exhibited a significantly more favorable course in the outpatient treatment group. Yet, at the 3 year follow-up, approximately one third of the patients in both treatment arms were still participating in their study treatments, with the potential for further improvement.

This study aimed to compare long-term outcomes in the two treatment groups using a wide range of clinical measures. More specifically, we addressed three research questions:

1. Are there differences in overall clinical course, from baseline to 6 years follow-up on the primary outcome variables, i.e., psychosocial functioning, interpersonal problems, symptom distress, and quality of life?

2. Are there any differences in patterns of clinical course, on the primary outcome variables between the 3- and 6-year follow-ups?

3. Are there any differences in secondary outcome variables at 6-year follow-up, i.e., diagnostic status, self-harm/suicidality, vocational functioning, and use of health care services?

## Methods

### Setting and design

The UPP was conducted at the Department of Personality Psychiatry (DPP) at Oslo University Hospital. The State Health Insurance Fund covered the expenses for both treatment conditions. Patients were evaluated before treatment, and after 8 months, 18 months, 3 years, and 6 years. All patients received optional psychopharmacological consultations with a psychiatrist as part of the follow-up evaluations. The staff at the DPP conducted the initial clinical and diagnostic evaluation, while PhD students and research assistants performed the follow-up interviews and diagnostic evaluations. Written informed consent was obtained from participants after they were provided with a description of the study. The Data Inspectorate and Regional Ethics Committee in Norway approved the project.

### Participants

Two hundred and fifty patients were referred to the DPP at Oslo University Hospital Ullevål during the intake period. Only patients with a diagnosis of PD were included. Exclusion criteria were schizotypal PD, antisocial PD, ongoing alcohol or drug dependence, psychotic disorders, bipolar I disorder, untreated attention deficit hyperactivity disorder (adult type), pervasive developmental disorder (e.g., Asperger’s syndrome), organic syndromes, and being homeless. One hundred and thirty-three patients were excluded because they had conditions listed in the exclusion criteria, did not have any PD, or did not attend the baseline evaluation. The patients’ pre-treatment demographics and clinical characteristics are reported in Table 1. After random assignment, there were no statistically significant differences in socio-demographic or clinical variables between the two groups, including the number of patients with axis I or axis II disorders and the mean number of axis II criteria.

**Table 1:** Baseline patient characteristics (n = 113)

	**Total (n = 113)**	**Step-down (n =59)**	**Outpatient (n = 54)**
Mean age (SD)	31 (7.3)	31 (7.2)	31 (7.4)
Women	75%	78%	72%
*Education and work status*			
More than 12 years of education	51%	47%	57%
Full-time employee (100%)	23%	23%	24%
Half-time employee (approx. 50%)	8%	12%	4%
Student	16%	16%	16%
Unemployed	50%	49%	50%
Other	3%	0%	6%
*Symptom severity*			
Mean symptom distress, GSI (SD)	1.72 (0.66)	1.71 (0.69)	1.75 (0.62)
Mean interpersonal problems, CIP (SD)	1.69 (0.51)	1.70 (0.53)	1.72 (0.48)
Mean psychosocial functioning, GAF (SD)	47.6 (4.4)	47.3 (4.3)	47.9 (4.6)
*Self-injury / suicide attempts*			
Suicidal thoughts in the last 7 days	50%	50%	50%
Suicidal thoughts in the last 12 months	85%	88%	82%
Self-injury in the last 12 months	31%	36%	26%
*Axis II diagnosis*			
Mean number PD diagnosis	1.4 (0.6)	1.5 (0.7)	1.4 (0.6)
Mean number PD criteria	15 (5.7)	15 (6.2)	15 (5.2)
Schizotype	0%	0%	0%
Schizoid	1%	2%	0%
Paranoid	14%	17%	11%
Histrionic	0%	0%	0%
Narcissistic	2%	0%	4%
Antisocial	0%	0%	0%
Borderline	46%	46%	46%
Avoidant	41%	44%	37%
Dependent	7%	7%	7%
Obsessive-compulsive	9%	12%	6%
PD NOS	21%	19%	24%
*Axis I diagnosis*			
Mean number axis I diagnosis (SD)	3.4 (1.4)	3.7 (1.3)	3.5 (1.5)
Mood disorders	87%	85%	89%
Anxiety disorders	87%	90%	83%
Substance abuse disorder	25%	19%	32%

### Completeness of data

**Figure 1:**
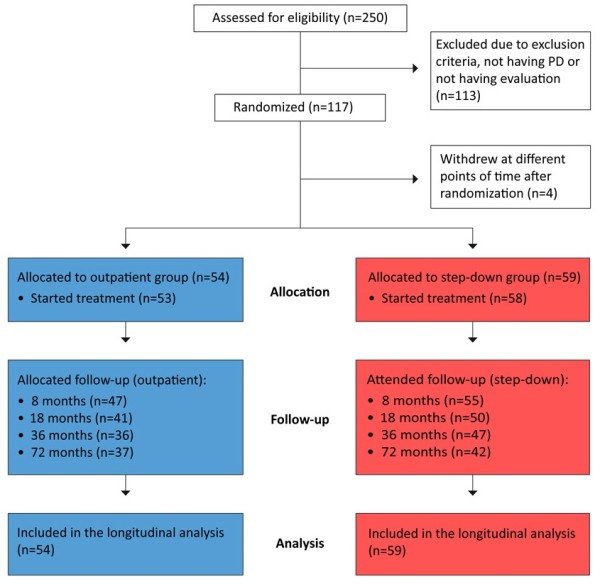
Patient dispensation in a randomized clinical trial comparing a step-down treatment program with outpatient treatment.

Figure 1 depicts the flow of patients throughout the study. After the initial evaluation, 117 patients were randomly allocated to one of the two treatment arms. Four patients withdrew from the study at different points after randomization. The present study included 113 patients: 59 in the step-down treatment arm and 54 in the outpatient treatment arm. Seventy-nine participants (70% of all participants) attended the 6-year follow-up investigation. These participants constituted 71% of the original participants in the step-down group and 67% of the original participants in the outpatient group (Figure 1). At the 6-year follow-up, there was no statistically significant difference in attendance rates between the two treatment groups. Moreover, there were no statistically significant differences in baseline variables between those who did and did not attend the 6-year follow-up analysis; however, there was a non-significant trend among those not attending towards having a higher score on the baseline Beck Depression Inventory (BDI) (p = 0.057). Fifty percent of the participants completed all five evaluations, while 4% attended only the baseline evaluation.

### Treatments

#### Step-down day hospital treatment (Step-down treatment)

The step-down day hospital treatment program consisted of an initial 18-week day hospital treatment phase, with a combination of psychodynamic and cognitive-behavioral group therapies for 3 to 4 days each week. The written treatment guidelines adhered to relational psychotherapy, with references to group analysis, self-psychology, and mentalization. After the initial day hospital treatment, the patients continued with outpatient combined psychotherapy. The outpatient treatment consisted of weekly group therapy (1.5 hours) for a maximum of 4 years, combined with weekly individual therapy for a maximum of 2.5 years. Fourteen percent of the patients dropped out of the step-down treatment before starting the combined outpatient psychotherapy. The average duration of treatments in the step-down group was 31 months (SD = 16), and the average number of outpatient consultations (counting both group and individual therapy) was 106 (SD = 76.0). The mean interval between the end of study therapy and the 6-year follow-up analysis (post-treatment phase) was 43 months (SD = 18).

#### Outpatient individual psychotherapy (Outpatient treatment)

The outpatient treatment consisted of different individual psychotherapies conducted mainly by therapists in private practice. The therapists were instructed to treat the patients according to their own preferred method and practice. The researchers gave no instructions to the therapists regarding the duration or intensity of psychotherapy, nor did they interfere with any treatment decisions. The average duration of treatment in the outpatient group was 24 months (SD = 20), and the average number of consultations was 56 (SD = 56.7). The mean interval between the end of study therapy and the 6-year follow-up analysis (post-treatment phase) was 47 months (SD = 20) in the outpatient treatment.

#### Therapists

To recruit individual therapists to the study, letters were sent to all therapists in Oslo with a contract with the State Health Insurance Fund, as well as professionals working in public mental health outpatient clinics. The therapists were assigned to one of the two treatment arms according to their own preferences, and signed a formal treatment contract with the project.

#### Group therapists in the step-down treatment

The 10 group therapists in the day hospital treatment were regular staff from the DPP (three psychiatric nurses, two psychiatrists, one residential doctor specializing in psychiatry, one specialist in clinical psychology, one art therapist, one social worker, and one physiotherapist). Seven of the 10 therapists had 5-years of training in group analysis. The mean age of the therapists was 48 years (SD = 9) and 80% were female. These therapists also conducted the outpatient group therapy.

#### Individual therapists in the step-down treatment

The individual therapists (n = 30) involved in the follow-up outpatient treatment included 16 psychologists, 12 psychiatrists, and two psychiatric nurses. Seven were recruited from the regular staff at the DPP. The therapists treated between one and three patients each. The mean age of the individual therapists was 50 years (SD = 9) and 57% were female. Their mean work experience as psychotherapists was 16 years (SD = 8). They were generally very satisfied with their work as therapists; when asked to rate their satisfaction on a scale ranging from 0 (no satisfaction) to 5 (very satisfied), the mean answer was 4.2 (SD = 0.6).

#### Therapists in the outpatient treatment

Thirty-two external therapists were recruited to provide outpatient treatment (16 psychologists, 15 psychiatrists, and one resident). Each therapist treated one to three patients. The mean age of these therapists was 55 years (SD = 8) and 41% were female. The mean work experience as psychotherapists was 20 years (SD = 8). The majority of these therapists practiced psychodynamic theoretical oriented therapy. At the same time, they reported being influenced by therapeutic theory and principles from other therapy models. They reported a mean of 4.0 (SD = 0.6) on the satisfaction scale. The individual therapists in the outpatient treatment arm were somewhat more experienced than the individual therapists in the step-down treatment arm (p < 0.05). The percentage of men was somewhat higher among the therapists in the outpatient group than among the individual therapists in the step-down group; however, this difference did not reach statistical significance.

### Outcome measures

#### Psychosocial functioning

Psychosocial functioning was rated using the Global Assessment of Functioning Scale (GAF) and the Work and Social Adjustment Scale (WSAS) [29]. The GAF is rated on a scale from 1 to 100, with a higher score indicating a higher level of functioning. The GAF scores were observer-rated according to a split symptom and function version [30]. Only the lowest GAF scores of symptoms and functions were used in the analyses. The staff at the DPP rated the GAF scores at baseline; while, research fellows conducted the GAF interviews at the 8-month, 18-month, 3-year, and 6-year evaluations. All raters were blinded concerning treatment conditions. The reliability of the GAF scores (intra-class correlation coefficient, ICC 2.1) was 0.56 at baseline, 0.81 at 8 months, 0.85 at 18 months, 0.94 at 3 years, and 0.92 at 6 years. The WSAS is a self-reported 5-item scale of functional impairment. It measures level of impairment on a scale from 0 to 8, where 0 indicates no impairment at all and 8 indicates very severe impairment. The scores on the five different items are totaled in a sum score (range: 0–40). Subjective Quality of Life (QoL) was assessed using a 10-point scale, with a score of 1 representing the lowest perceived QoL and a score of 10 indicating the highest perceived QoL.

#### Interpersonal problems

Self-reported interpersonal problems were assessed by the Circumplex of Interpersonal Problems (CIP) [31], a 48-item Norwegian version of the Inventory of Interpersonal Problems-Circumplex version (IIP-C) [32] with a 5-point Likert response format ranging from 0 to 4. A higher score indicates more interpersonal problems. The sum scores of the two versions correlate at a level of 0.99.

#### Symptom distress

Self-reported symptom distress was measured by the Symptom Checklist, SCL-90-R [33]. The SCL-90-R was designed to cover the major symptoms of psychiatric distress, summarized in a Global Severity Index (GSI) with scores ranging from 0 to 4, with higher scores indicating more symptom distress. Characteristic attitudes and symptoms of depression were assessed using the BDI [34]. Sum scores between 19 and 29 indicate moderate depression, while scores ≥30 indicate severe depression.

#### Axis I and II diagnoses

Axis I and II diagnoses were based on the Mini International Neuropsychiatric Interview (MINI) [35] and the Structured Clinical Interview for DSM-IV (SCID-II) [36], respectively. At the 6-year follow-up, 30 videotapes were evaluated by two independent raters. The reliability coefficients were (ICC 2.1) were 0.76, 0.87, and 0.92 for the number of axis I diagnoses, number of axis II diagnoses, and total number of SCID-II criteria, respectively. The diagnostic reliability at baseline and at the 3-year follow-up were also acceptable and were reported previously [28].

#### Self-harm, suicidal thoughts and suicide attempts

Incidents of self-harm and suicide attempts were self-reported by patients and then confirmed during the research interviews. At baseline and at the 6-year follow-up, the patients were asked to report self-harm and suicide attempts during the last 12 months. When assessed at the 8-month, 18-month, and 3-year follow-ups, patients were asked to report incidents since the last follow-up, as well as suicidal thoughts during the last seven days.

#### Statistical analysis

All results were analyzed using an intention-to-treat approach based on treatment assignments. Longitudinal analysis was used to assess change over time and relate the changes to covariates, particularly treatment group assignment. We used a linear mixed model (LMM) for the continuous outcomes, with maximum likelihood as the method of estimation. Piecewise linear splines, with one knot at 3-years for all outcomes, were used to describe the main features in the observed outcomes. Separate random intercepts and slopes were included when proven to enhance the model fit. The parameters of main interest were the fixed effect interaction terms between groups and times, prior to and following the knot, describing whether the patients in the two groups changed differently across the observation period. Assumptions about withdrawal from follow-up evaluations (missing at random, MAR) and selection bias follow descriptions in previous reports [28]. Although it is not possible to test the MAR assumption, a comparison with a complete case analysis (participants without dropouts) is informative and was conducted in this study. When interpreting the significance levels, it is important to keep in mind that no correction for multiple testing was conducted in this trial. Residual analyses and a search for outliers were performed to assess model adequacy. The treatment effects at the 6-year follow-up on axis I and II diagnoses were compared using an independent *t*-test (two-tailed) and chi-squared statistics. For the analysis of vocational functioning and the estimates of health care use, the normal distribution range was not fulfilled and non-parametric tests were used. Within-group pre-post effect sizes were computed using Cohen’s d, with pooled pre- and post-SD adjustment for sample size. All analyses were performed using SPSS (version 18; SPSS Inc.).

## Results

### Overall clinical course from baseline to the 6-year follow-up

According to the LMM analyses, there were no statistically significant differences between treatment groups at the 6-year follow-up for the primary outcome variables. The p-value of the different outcomes between treatment conditions were: GAF (p = 0.52), WSAS (p = 0.47), CIP (p = 0.96), GSI (p = 0.38), BDI (p = 0.47), and QoL (p = 0.27). Both groups improved on all outcome variables between baseline and 6 years. The means and SDs for the main outcome measures, as well as the within-group effect sizes are shown in Table 2. The mean effect size (*d*) was 1.07 (range: 0.56-1.92) in the step-down group from baseline to 6 years and 0.87 (range: 0.66-1.10) in the outpatient group.

**Table 2:** Mean outcome measures (with SD) and effect sizes * (Cohens’ ***d*) of the step-down and outpatient treatments at baseline and at 8, 18, 3, and 6- years of follow-up**

**Step-down**											
	**Baseline**		**8 months**		**18 months**		**3- years**		**6- years**		***d***
GAF	47.3	(4.3)	51.5	(10.2)	53.5	(10.3)	57.7	(12.7)	62.7	(13.6)	1.92
WSAS	25.3	(7.2)	20.6	(8.6)	19	(9)	19.2	(10.6)	13.7	(11.0)	1.19
GSI	1.71	(0.69)	1.43	(0.65)	1.32	(0.75)	1.19	(0.75)	0.91	(0.72)	1.14
BDI	18.3	(8.2)	17.2	(7.6)	15.5	(10.7)	14.7	(11.3)	13,0	(11.2)	0.56
CIP	1.66	(0.53)	1.53	(0.59)	1.51	(0.6)	1.42	(0.62)	1.13	(0.68)	0.89
QOL	3.3	(1.6)	4.5	(1.6)	4.9	(2.1)	5.4	(2)	6.1	(2.3)	1.49
PDcrit	13.3	(5.6)					8.5	(6.4)	5.9	(6.5)	1.24
**Outpatient**											
	**Baseline**		**8 months**		**18 months**		**3- years**		**6- years**		***d***
GAF	47.9	(4.6)	50.2	(12)	56.8	(11.7)	66.9	(12.6)	57.1	(14.5)	1.10
WSAS	24.7	(8.2)	18.9	(9.9)	15.7	(11.2)	12.6	(10.9)	18.3	(11.3)	0.68
GSI	1.75	(0.62)	1.43	(0.73)	1.23	(0.73)	1.06	(0.79)	1.12	(0.74)	0.95
BDI	19.9	(9.5)	18.5	(11.8)	16.6	(12.5)	12.2	(10.3)	13.4	(9.95)	0.66
CIP	1.72	(0.48)	1.52	(0.57)	1.25	(0.62)	1.22	(0.64)	1.32	(0.62)	0.75
QOL	3.6	(1.7)	4.4	(1.9)	5.1	(2.3)	5.9	(2.2)	5.5	(1.85)	1.08
PDcrit	13.4	(4.8)					6.0	(5.8)	5.6	(5.6)	1.53

Note. *GSI*: Global Severity Index; *GAF*: Global Assessment of Functioning; *CIP*: Circumplex of Interpersonal Problems; *BDI*: Beck depression inventory; *WSAS*: Social and Adjustment Scale; *QoL*: Quality of Life. *Effect sizes (*d*) are pre-post estimates from baseline to 6- years of follow-up.

### Clinical course from the 3- to 6-year follow-up

**Figure 2:**
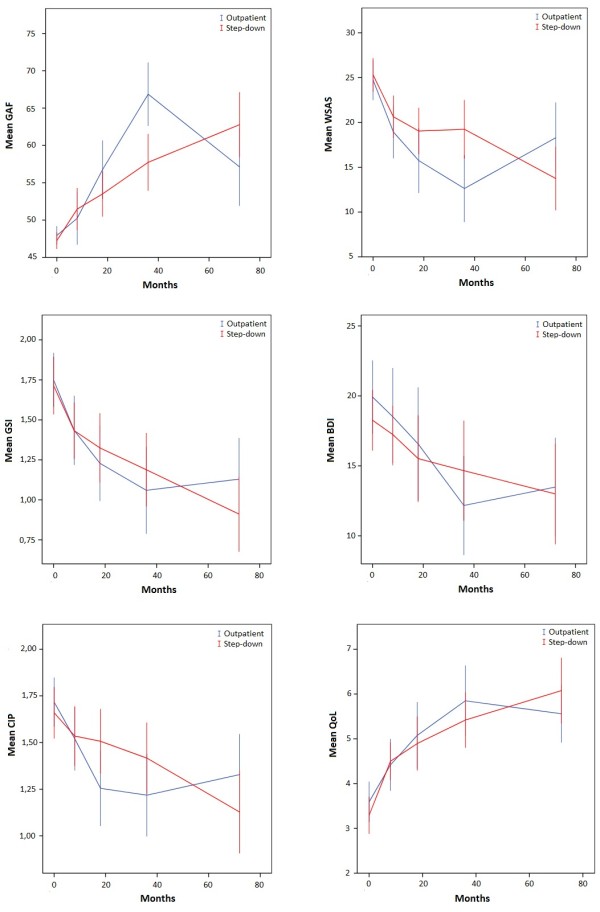
Course of primary outcome variables.

The LMM analyses revealed statistically significant differences in the course of psychosocial functioning (Figure 2 and Table 3). Patients in the outpatient treatment arm experienced a significantly greater increase in psychosocial functioning between 18 months and 3 years [28]. However, during the period from 3–6 years, the level of functioning was reversed in the outpatient group. In contrast, there was stable growth in the level of functioning in the step-down group. This between-group difference was statistically significant for both GAF (p < 0.001) and WSAS (p = 0.001), favoring the step-down treatment. For the other outcome measures, the interaction between treatment group and time (3–6 years) was not statistically significant, although the difference in the course of the CIP trended towards significance (p = 0.055) in favor of the step-down program.

**Table 3:** Fixed effects estimates in the linear mixed model for psychosocial function from baseline to 3 years and from 3–6 years

**Outcome**	**Fixed effects**	**Estimates**^**1**^	**SE**	**95% CI**
GAF	*Main effect group*			
	Outpatient group	0.63	0.86	-1.07, 2.33
	Step-down group	0 (Ref)		
	*Main effect time*			
	Time ≤ 3- years	0.28***	0.05	0.18, 0.37
	Time > 3- years	0.14*	0.06	0.14, 0.26
	*Interaction, group x time ≤ 3- years*			
	Outpatient group	0.21**	0.73	0.06, 0.36
	Step-down group	0 (Ref)		
	*Interaction, group x time > 3- years*			
	Outpatient group	-0.38***	0.10	-0.67, -0.18
	Step-down group	0 (Ref)		
WSAS	*Main effect group*			
	Outpatient group	-1.50	1.53	-4.55, 1.55
	Step-down group	0 (Ref)		
	*Main effect time*			
	Time ≤ 3- years	-0.06	0.03	-0.12, 0.01
	Time > 3- years	-0.14*	0.04	-0.23, 0.05
	*Interaction, group x time ≤ 3- years*			
	Outpatient group	-0.10	0.05	-0.20, 0.004
	Step-down group	0 (Ref)		
	*Interaction, group x time > 3- years*			
	Outpatient group	0.25***	0.07	0.11, 0.38
	Step-down group	0 (Ref)		

Note. *GAF*: Global Assessment of Functioning; *WSAS*: Work and Social Adjustment Scale.

^1^The time unit is one month. *p ≤ 0.05; **p ≤ 0.01; ***p ≤ 0.001.

### Secondary outcome variables at the 6-year follow-up

There were no statistically significant differences between the two treatment groups at the end of the follow-up period with respect to the numbers of patients with axis I or axis II disorders. The step-down group had a mean number of 1.7 (SD = 1.5) axis I diagnoses, versus 2.3 (SD = 2) in the outpatient group. Thirty percent of the participants at the 6-year follow-up had been diagnosed with at least one PD. The mean number of PDs was 0.45 (SD = 0.75) in the step-down group and 0.31 (SD = 0.59) in the outpatient group. The mean number of PD criteria met was 5.8 (SD = 6.5) in the step-down group and 5.6 (SD = 5.6) in the outpatient group.

At the 6-year follow-up, 10% of patients in the step-down group and 12.5% of patients in the outpatient group reported self-harming behavior in the last 12 months. Twenty-one percent of patients in both treatment arms reported suicidal thoughts in the past 7 days. Forty percent of patients in the step-down group and 41% of patients in the outpatient group reported suicidal thoughts in the past 12 months. One patient in the step-down group reported a suicide attempt during the past 12 months, while no suicide attempts were reported in the outpatient group. One patient in the step-down group committed suicide 9 months after withdrawing from the day hospital treatment. According to the Norwegian national registration office, two more patients (from the outpatient treatment arm) had died. The causes of these patients’ deaths are unknown.

At the 6-year evaluation, 61% of participants had worked or studied at some point during the past year: 67% in the step-down group and 54% in the outpatient group. The median number of months spent working or studying was 7 (range: 0–12) in the step-down group and 2 (range: 0–12) in the outpatient group. This difference was not statistically significant. However, 63% of patients in the step-down group and 34% of patients in the outpatient group had a GAF score >60 (p = 0.018). The use of health care services, like contact with a general practitioner and current outpatient treatment, did not differ significantly between groups (Table 4). The complete case analyses showed a similar pattern as the intention to treat analyses. According to the complete case analyses, there was a statistically significant difference in the course of psychosocial functioning, as assessed by GAF and WSAS, favoring the step-down treatment in the 3 to 6 year period.

**Table 4:** Use of health services during the past 12 months, at 6- years of follow-up

	**Total (n = 79)**	**Step-down (n =42)**	**Outpatient (n = 37)**
Patients with GP visits	92%	93%	92%
Patient visiting GP for mental health issues	57%	50%	65%
Median number of GP visits	4	4	5
Currently receiving psychiatric treatment*	48%	44%	53%
Outpatient treatment	29%	21%	38%
Contact with psychiatric emergency	3%	2%	3%
Contact with a psychiatric nurse	11%	10%	14%
Substance addiction clinic	5%	2.5%	3%
Regular psychiatric medication	43%	33%	54%

*Includes any form of psychiatric treatment (e.g., regular contact with a psychiatric nurse, psychiatric medication, regular psychotherapy, or day treatment at a psychiatric hospital).

## Discussion

### Summary of findings

First, there were no statistically significant differences between the treatment groups regarding the primary outcome variables from baseline to the 6-year follow-up. Both groups exhibited clinical improvement during the 6-year period. The effect sizes were mainly in the large range for both treatment groups; although, there was a tendency towards larger effect sizes in the step-down treatment arm. Second, patients in the outpatient group had a marked decline in psychosocial functioning during the period between the 3- and 6-year follow-ups; while, psychosocial functioning continued to improve during the same period in the step-down group. This effect was statistically significant. Finally, 30% of patients still had a PD diagnosis at the 6-year follow-up, with no significant differences between the two treatment arms. There were also no statistically significant differences between groups concerning self-harm/suicidality, vocational functioning, and use of health care services.

### Comparison with other studies

The findings in the present study differ from those of other step-down programs by Chiesa et al. [18] and Fonagy and Bateman [19], where superior outcomes were observed for the step-down treatment. Some possible explanations for these differences should be discussed. First, there is considerable variety among the programs with regard to their duration, intensity, and composition of their respective treatment elements. Chiesa et al. [18] investigated a model consisting of a 6-month inpatient stay followed by 12–18 months of outpatient combined group and individual treatment; while, the study by Fonagy and Bateman included 18 months of mentalization-based day hospital treatment followed by group therapy twice weekly for a maximum of 18 months [37]. In the present study, the initial phase was restricted to 18 weeks of day hospital treatment. This was followed by the long-term outpatient therapy consisting of weekly combined group and individual psychotherapy. Second, there were notable differences in comparison groups. Chiesa et al. compared their step-down model to groups receiving long-term residential treatment or general psychiatric community treatment. Also, it should be mentioned that the allocation to different treatment conditions was not based on randomized. The comparison group in Fonagy and Batemans’ study received treatment as usual in the local community. Thus, neither of these studies included outpatient individual therapy as a comparison group. It is difficult to make direct comparisons between the studies due to these differences in program characteristics and control groups. Also, the present study included a mixed PD sample, although schizotypal and antisocial PDs were excluded. It is not clear whether step-down models suit certain PD categories (e.g., borderline PD) more than others and this should be investigated further [16]. We also need to know more about how different levels of PD severity can affect treatment outcome, e.g. whether patients in the most severe end of the severity spectrum will profit from more or less intensive treatment or psychosocial support. For instance, Chiesa [38,39] found that more severe PD patients seemed to respond better to a less intensive community based treatment regime than an intensive long-term residential stay.

The present finding of equal long-term outcomes should be considered in light of accumulating evidence of the benefits of outpatient individual therapy for patients with PDs, particularly borderline PD [20,40,41]. Also, such benefits are not limited to specialized manualized therapies. McMain [42] and Vinnars [43] compared non-manual based treatments with manual based therapies in community and outpatient settings and found their effects were similar. In the UPP, the patients in the outpatient treatment arm received non-manualized psychotherapy from therapists who were instructed to treat the patients in accordance with their usual practice.

### Clinical considerations

The present findings suggest that both hospital-based long-term step-down treatment and outpatient individual psychotherapy with therapists with the opportunity to practice long-term treatment may help improve symptoms and psychosocial in poorly functioning PD patients. Interestingly, analyses of treatment costs from baseline to the 3-year follow-up showed no significant differences in total treatment costs between the treatment conditions when both costs related to study treatment and additional psychiatric treatments were included [44]. The present study did not include cost analyses; however, there were small differences in the use of health care services at the 6-year follow-up, which suggests the differences in the overall costs between the treatment programs are minor.

Taking into account the superior psychosocial outcome for the outpatient treatment condition at the 3- year follow-up, the divergence in the pattern of the clinical course between the treatment groups from the 3- to 6-year follow-ups was unexpected. The finding at the 6-year follow-up is strengthened by the observer-rated GAF values and self-rated WSAS scores, which both demonstrated the same statistically significant patterns between 18 months and 3 years and between 3 and 6 years. Moreover, the shift in the proportion of patients with GAF levels >60 further supports this pattern. A GAF score <60 is descriptive of moderate to severe psychosocial dysfunction, and a GAF score >60 indicates a range from mild dysfunction to a high level of functioning. At the 3-year evaluation, only one-third of patients in the step-down group and two-thirds of patients in the outpatient group had a GAF level >60 [28]. In contrast, at the 6-year follow-up, two-thirds of the step-down group and one-third of the outpatient group had a GAF level >60. This between-group difference was not only statistically significant, but also clinically significant.

Some plausible explanations concerning the divergence in clinical course between groups should be discussed. First, group therapy was an essential element in the step-down condition. Group therapy is a demanding format for patients with PDs that activates and challenges their interpersonal problems and emotional difficulties. The gains may be harder to reach and slower to appear when struggling with such core issues over time in therapy groups. However, if sufficiently integrated, the experiences might serve as a basis for further development after the end of treatment. Next, the step-down program implied a disruption of attachments to therapists and group members after a short intensive day treatment phase, and required that the patients form new emotional bonds with group members and therapists in outpatient treatment. The attachment difficulties typical of patients with severe PD make those patients vulnerable during transitions between therapies. This vulnerability may have affected their therapeutic process and caused a delay in clinical improvement [45]. In fact, this was the view of the clinical management, who decided to close the step-down program in 2008 because of the results of this study. The program was changed to an outpatient mentalization-based treatment program without any day hospital component. Conversely, the outpatient group might have benefitted from the possibility of a stable and continuing attachment relation to one therapist during the entire treatment period, and thereby achieved faster improvement in psychosocial adaption. Yet, the advantage observed at the 3-year follow-up was not sustained after 6 years. At the 6-year follow-up, most patients had been in the post-treatment phase for a long time. One may speculate whether the less intensive outpatient individual therapy had a more supportive function in relation to psychosocial problems, which facilitated social adjustment in the short run for certain patients but was easier lost in the post-treatment phase. Finally, treatment received after secession of UPP treatment could potentially explain the differences in clinical course between treatment arms. However, there were no significant differences in health care services received in the past year between groups at the 6-year follow-up.

### Strengths and limitations

The strengths of the present study are the randomized design and the extended follow-up of long-term therapies for patients with PDs. At present, both step-down models and outpatient therapies are realistic treatment options for the majority of dysfunctional PD patients, and comparisons between treatments at such different levels of care are needed. The day hospital part of the step-down program was quite representative for short-term day hospitals in Norway [11]. Moreover, the two treatments were conducted in ordinary clinical settings and only a few severe cases with complicated comorbidities were excluded, which strengthens the external validity of the study. High dropout rates and unstable attendance are common in the treatment of patients with PDs, and high attrition from follow-up evaluations may be a challenge in longitudinal studies of this patient group. The response rate at the 6-year follow-up was approximately 70%, which is considered satisfactory. Certain limitations of the present study should be noted, however. First, as outlined above, missing data and violation of the MAR assumption could bias the results. However, complete case analyses support the validity of the present findings. In addition, in the present study we did not control for other kinds of treatments that patients may have received during the 6-year follow-up period. Data from the 3-year follow-up showed that treatment outside the UPP was common for certain patients [44]. Finally, the study did not take into account or model the possible influence of life events or other extra-therapeutic circumstances.

## Conclusion

The findings indicate that both hospital-based long-term step-down treatment and outpatient individual psychotherapy with therapists who are able to provide long-term treatment may help improve symptoms and psychosocial functioning in poorly functioning PD patients, although patients with schizotypal and antisocial PD were not included in this study. The step-down group continued to improve in social and interpersonal functioning in the post-treatment phase, suggesting that longer-term changes were stimulated during treatment. In concordance with most treatment studies of patients with PDs, there was considerable variation in outcome among the patients across treatments. We need to further improve our understanding of various patient and treatment characteristics that might influence the long-term course of these patients, and whether some treatments work better for certain patients.

## Competing interests

The authors declare that they have no competing interests.

## Authors’ contributions

TW, SK, ØU, and GP were responsible for designing the UPP-study and selecting study instruments. MSJ, BH, and AK contributed to the acquisition of data. BTA and TW analyzed the data, with statistical contributions by OK. The manuscript was drafted by BTA and TW. EHK and BH contributed to the interpretation of results. All authors reviewed, revised, and approved the final version of the manuscript.

## Pre-publication history

The pre-publication history for this paper can be accessed here:

http://www.biomedcentral.com/1471-244X/14/119/prepub
